# Cerebrospinal fluid analysis: current diagnostic methods in central nervous system infectious diseases

**DOI:** 10.1590/0004-282X-ANP-2022-S114

**Published:** 2022-08-12

**Authors:** Hélio Rodrigues Gomes

**Affiliations:** 1 Universidade de São Paulo, Hospital das Clínicas, Divisão de Laboratório Clínico, Laboratório de Líquido Cefalorraquidiano, São Paulo SP, Brazil.; 2 Universidade de São Paulo, Departamento de Neurologia, São Paulo SP, Brazil.

**Keywords:** Cerebrospinal Fluid, Central Nervous System Bacterial Infections, Biomarkers, Metagenomics, Neurosyphilis, Meningoencephalitis, Líquido Cefalorraquidiano, Infecções Bacterianas do Sistema Nervoso Central, Biomarcadores, Metagenômica, Neurossífilis, Meningoencefalite

## Abstract

Cerebrospinal fluid (CSF) analysis is an important diagnostic tool for many conditions affecting the central nervous system (CNS), especially CNS infectious diseases. Despite its low specificity, CSF white blood cell counts, CSF protein levels, CSF serum glucose ratio and CSF lactate measurement are useful in differentiating infections caused by distinct groups of pathogens. CSF direct examination and cultures can identify causative organisms and antibiotic sensitivities as well. Adjunctive tests such as latex agglutination, different immunological assays and molecular reactions have great specificities and increasing sensitivities. In this article, some recent diagnostic methods applied to CSF analysis for frequent CNS infections are presented.

## RESUMO

A análise do líquido cefalorraquiano (LCR) é uma importante ferramenta diagnóstica para muitas condições que afetam o sistema nervoso central (SNC), especialmente as doenças infecciosas. Apesar da baixa especificidade, a contagem de leucócitos no LCR, a determinação dos níveis de proteína, glicose e lactato podem ser úteis na diferenciação de infecções causadas por diferentes grupos de patógenos. O exame direto e as culturas podem identificar organismos causadores de infecções bem como suas sensibilidades a antibióticos. Testes adjuvantes como aglutinação em látex, diferentes ensaios imunológicos e reações moleculares têm taxas de sensibilidades e especificidades crescentes. Neste artigo, são apresentados alguns métodos diagnósticos mais recentemente aplicados à análise do LCR no diagnóstico das infecções do SNC.

## INTRODUCTION

Since the end of the eighteenth century, cerebrospinal fluid (CSF) analysis has been used as an important diagnostic tool for many conditions affecting the central nervous system (CNS), especially CNS infectious diseases. A lumbar puncture (LP) is an invasive technique that accesses the restricted compartment of the subarachnoid space in order to sample CSF. This procedure involves introducing a needle below the termination of the spinal cord, passing through the dura mater of the spinal cord, and permitting access to the subarachnoid space. LP indications: Quincke described an LP in 1891, being used therapeutically to relieve increased intracranial pressure in children with meningitis. LP is contraindicated if the risk of the procedure outweighs the potential benefit. Clinical contraindications to LP include anticoagulation, clinical evidence of disseminated intravascular coagulation and local infection or loss of skin integrity at the puncture site (Table 1). Table 2 presents conditions that indicate CT scan prior to LP^1^,^2^. Despite having low specificity, CSF white blood cell counts, CSF protein levels, CSF serum glucose ratio and CSF lactate measurement are useful in differentiating infections caused by distinct groups of pathogens. CSF direct examination and cultures can identify causative organisms and antibiotic sensitivities. Adjunctive tests such as latex agglutination, different immunological assays and molecular reactions have great specificities and increasing sensivities^2^.

**Table 1.** Contraindications for lumbar puncture.

Platelet count < 40.000/mm³	International normalized ratio (INR) <1.5	Local skin infection	Local developmental abnormality, e.g., myelomeningocele	Raised intracranial pressure (with a pressure gradient across the CNS compartments)

**Table 2.** for CT head prior to LP.

	IDSA guideline	ESCMID guideline
Immunosuppression	HIV infection, immunosuppressive therapy, post-transplantation	Severely immunocompromised state
Background of CNS disease	Mass lesion, stroke or focal CNS infection	No recommendation
Seizures	Seizures within a week prior to presentation	New onset seizures
Level of consciousness	GCS < 15	GCS < 10
Focal neurological deficit	Focal deficit including cranial nerve palsies	Focal deficit excluding cranial nerve palsies
Papilloedema	Indication for CT	No recommendation
Duration of symptoms	No recommendation	No recommendation

BP: blood pressure; CNS: central nervous system; CT: computed tomography; ESCMID: European Society of Clinical Microbiology and Infectious Diseases; GCS: Glasgow Coma Scale; HIV: human immunodeficiency virus; IDSA: Infectious Diseases Society of America; LP: lumbar puncture.

## ACUTE AND CHRONIC MENINGITIS AND MENINGOENCEPHALITIS

CSF analysis is of vital importance in suspected meningitis as clinical characteristics alone are unable to distinguish meningitis from other diagnoses, and bacterial from non-bacterial etiologies. For the majority of patients who do not require CT prior to lumbar puncture (LP) and do not have another clinical contraindication to LP, CSF analysis should be performed as soon as possible, before CSF is rendered sterile by broad-spectrum antibiotics^3^ ^,^ ^4^.

CSF leukocyte count is often helpful in distinguishing bacterial from non-bacterial meningitis. Meningitis is confirmed when the leukocyte count in the CSF exceeds 5 cells/μL. A leukocyte count of ≥1000 cells/μL with a neutrophilic predominance is highly suggestive of bacterial meningitis, whereas <1000 cells/μL with a lymphocytic predominance is more consistent with viral meningitis, tuberculous meningitis (TBM) or cryptococcal meningitis. Bacterial meningitis due to*L. monocytogenes*is an important exception, with approximately 60% of cases having a leukocyte count of <1000 cells/μL, which may be lymphocytic. CSF protein is usually elevated in meningitis of both bacterial and viral etiology due to increased permeability of the blood-brain barrier as a consequence of inflammation. This elevation is typically greater in bacterial and TBM than in viral meningitis, with the exception of meningitis due to the herpes simplex virus (HSV) or varicella zoster virus (VZV). Low CSF glucose is another helpful pointer towards bacterial meningitis, although it is also seen in TBM and cryptococcal meningitis. CSF glucose varies proportionally to blood glucose, with a normal CSF-to-blood glucose ratio being 0.6. A ratio of ≤0.5 has 100% sensitivity for bacterial meningitis, but specificity of only 57%. When a ratio of ≤0.23 is used, specificity improves to 99%, but at the cost of sensitivity. The CSF-to-blood glucose ratio may be less helpful in hyperglycaemic patients. CSF lactate is helpful in distinguishing bacterial from non-bacterial meningitis. A meta-analysis reported that a CSF lactate of ≥35 mg/dL had 93% sensitivity and 99% specificity for bacterial meningitis^3^ ^,^ ^5^.

The likelihood of a positive Gram stain ranges from 25% to 97% and is highly correlated with the concentration of bacterial colony-forming units in the CSF; a negative stain therefore cannot exclude this diagnosis. A positive Gram stain is more likely in pneumococcal meningitis compared with meningococcal or*Listeria*meningitis and is less likely if antibiotics have been given prior to LP^6^. 

Multiplex PCR assays can detect the presence of many important bacterial and viral pathogens in CSF, including*N. meningitidis, S.pneumoniae,*enteroviruses, HSV, VZV and mumps virus, with sensitivity and specificity ≥ 90%. Misleading positive viral results have occasionally been described in cases of confirmed bacterial meningitis. When compared with bacterial culture, PCR is much less affected by pretreatment with antibiotics. PCR has been used successfully for the diagnosis of*Listeria*meningitis, but assays are not yet widely available. The FilmArray meningitis/encephalitis panel (Biofire, Salt Lake City, UT) is a multiplex PCR assay that detects 14 meningitis-causing pathogens (bacteria, viruses, and fungi)^7^ ^-^ ^9^.

Recent data suggest that while small multiplex panels targeting *H influenzae*, meningococci and pneumococci are highly sensitive and specific, larger panels that include viral, nosocomial and rarer community-acquired pathogens have varying sensitivity and specificity and are not currently recommended. The multiplex PCR assay (FilmArray® meningitis/encephalitis multiplex PCR assay) reduces time to microbiological diagnosis by 3.3 ± 1.6 days and allows an earlier discontinuation of empirical anti-infective drugs and an earlier hospital discharge^10^ ^,^ ^11^.

More recently, direct next-generation sequencing (NGS) and metagenomics of CSF have been proposed to detect pathogens in cases with a high index of clinical suspicion of acute meningitis, but negative PCR tests. Like culture, PCR, and antigen-based testing, metagenomic NGS is fundamentally a direct-detection method and relies on the presence of nucleic acid from the causative pathogen in the CSF sample. While this approach is promising, constraints around cost, bioinformatics expertise and clinically relevant turnaround times have limited clinical use of NGS to date, as discussed later^12^ ^,^ ^13^.

As subacute and chronic meningitis are diagnostically challenging given the wide range of potential infectious, autoimmune, neoplastic, paraneoplastic, parameningeal, and toxic causes CSF metagenomics NGS can be more useful in establishing the etiologic diagnosis. Wilson et al. studying seven patients with subacute or chronic meningitis identified a parasitic worm, a virus, and 4 fungi by CSF metagenomic NGS^14^.

The same group analyzed^13^ the clinical usefulness of metagenomic NGS for diagnosing neurologic infections in a series of patients with idiopathic acute meningitis, encephalitis, or myelitis at the time of enrollment, in parallel with conventional microbiological testing. It was observed that metagenomic NGS assay identified more potential pathogens than conventional direct-detection testing of CSF (32 vs. 27). Their findings suggested that a negative test should be interpreted with caution owing to the higher risk of false negative results. However, metagenomic NGS assay performed in combination with conventional testing may potentially be useful for ruling out an active infection in patients with suspected autoimmune encephalitis, who typically present with only mild-to-moderate lymphocytic pleocytosis (<100 cells per cubic millimeter) and thus low host background in CSF^13^.

## FUNGAL MENINGITIS

Cryptococcal meningitis causes 15% of AIDS-related deaths, and C. neoformans is an important agent related to meningitis in other immunocompromised conditions and should be considered in immunocompetent patients. A high opening pressure is characteristic of this condition, mainly in HIV patients, and the diagnosis can be confirmed through CSF analysis for cryptococcal antigen (CrAg), India ink staining and fungal cultures, which are positive in 97%, 51% and 89%. CrAg results can be qualitative, or semiquantitative with titers by 1:2 serial dilution but the process of performing the test requires testing in a laboratory environment, and thus skilled laboratory workers, steady electricity, heat inactivation, cold-chain shipping, and refrigeration of reagents, that is feasible in high-income country laboratories but not in countries where the majority of cryptococcal infection occurs. Cheaper and easier to be performed CrAg lateral flow assay (LFA) is an immunochromatographic dipstick assay that also detects antigen with qualitative or semiquantitative results in serum, plasma, or CSF samples. Studies demonstrate sensitivity of 99.3% and specificity of 99.1% for CSF^15^. Other available diagnostics techniques include PCR (96% sensitivity and 100% specificity) and matrix-assisted laser desorption ionization-time-of-flight mass spectrometry (MALDI-TOF) ^16^.

## TUBERCULOUS MENINGITIS

Tuberculous meningitis (TBM) can present as either an acute or subacute illness and should always be considered in confusional states and consciousness impairment, mainly in immunocompromised subjects. TBM remains difficult to diagnose and the typical CSF pattern (elevated protein, lymphocytic pleocytosis, and low glucose) cannot reliably distinguish TBM from other forms of sub-acute meningitis. Traditional tests to detect bacilli include acid fast bacilli (AFB) smear and culture, whose sensitivities are low (10-15% and 50-60%, respectively) and long result returns do not allow prompt clinical intervention^17^.

The CSF contains a plethora of metabolic information that can be used to describe TBM. The two primary metabolic markers that consistently arise from CSF metabolomics studies of TBM are CSF lactate and CSF glucose Collectively, CSF metabolomics studies have identified five classes of metabolites that characterize TBM: amino acids, organic acids, nucleotides, carbohydrates, and “other”^18^.

In recent years, nucleic acid amplification tests (NAATs) have been utilized for TBM diagnosis. The most notable tests are GeneXpert MTB/RIF (Xpert) and the re-engineered GeneXpert MTB/RIF Ultra (Xpert Ultra). In 2013, the WHO recommended Xpert as the initial test for diagnosis of TBM based on a systematic review of 13 studies. Sensitivity is generally similar to culture (50-60%), but with a run-time of two hours. Xpert and culture frequently detect cases that the other modality misses and Xpert’s negative predictive value is only 84-94%, meaning that while Xpert is helpful if positive, it cannot effectively rule out TBM. One way to maximize Xpert’s utility is to centrifuge larger volumes of CSF (>5ml). Xpert Ultra utilizes the same instrument as Xpert with a software upgrade. WHO has recommended Xpert Ultra in place of Xpert for TB meningitis based on one study of Ugandan HIV-infected adults with suspected TBM, which found 95% sensitivity versus a composite microbiological end point, and 70% sensitivity versus consensus research case definitions. A recent cohort reported 86% sensitivity for Xpert Ultra for definite TBM versus 36% for Xpert and 14% for culture. Negative predictive values remain inadequate to ‘rule-out’ TBM and the publication of additional studies of Xpert Ultra is required to fully evaluate this technology. Recently it was observed that test sensitivities were 76.5% (95% CI, 62.5-87.2%) for Xpert Ultra, 55.6% (95% CI, 44.0-70.4%) for Xpert, and 61.4% (95% CI, 45.5-75.6%) for mycobacterial culture^19^.

## NEUROSYPHILIS

**Figure 1.**
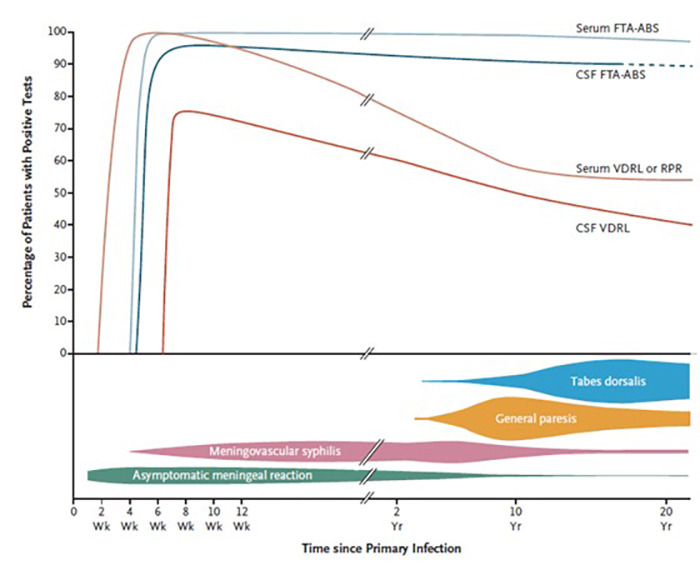
Laboratory and clinical features of neurosyphilis according to infection time. The dashed line for de FTA-ABS test indicates uncertain test results.

The diagnosis of symptomatic neurosyphilis requires meeting clinical, serologic, and cerebrospinal fluid (CSF) criteria, while the diagnosis of asymptomatic neurosyphilis relies on serologic and CSF criteria alone. CSF abnormalities include pleocytosis, elevations of protein level and gamma globulin percentual and reactivity of nontreponemal tests (VDRL and RPR) and treponemal tests (fluorescent treponemal antibody absorption - FTA-ABS - and agglutination techniques). As T. pallidum IgG can cross the intact blood-CSF barrier, reactive treponemal tests in the CSF are not specific for the diagnosis of neurosyphilis. A CSF positive VDRL test points out the definitive diagnosis. Agglutination techniques and CSF FTA-ABS have similar sensitivity and specificity and a negative CSF treponemal test may not rule out neurosyphilis among patients with a high pretest probability (patients with syphilis and neurologic symptoms)^20^,^21^. Figure 1 shows laboratory and clinical features of neurosyphilis and Table 3 presents the sensitivities and specificities of different CSF parameters in neurosyphilis. 

*Apud* Ropper AH.

FTA-ABS: fluorescent treponemal antibody absorption; CSF: cerebrospinal fluid; VDRL: venereal disease research laboratory; RPR: rapid plasm regain; Wk: week; Yr: year.

**Table 3.** Sensivity and specificity of neurosyphilis tests according to clinical evolution. *Apud* Ropper AH.

Test	Sensitivity (%)		Specificity (%)
	Early neurosyphilis	Late symptomatic neurosyphilis	Late symptomatic neurosyphilis
CSF VDRL	75	30-70	100
CSF FTA-ABS	100	~ 99	50-70
White-cell count > 5-10/mm³	100	95	~97
Protein > 45 mg/dL	90	95	< 50

FTA-ABS: fluorescent treponemal antibody absorption; CSF: cerebrospinal fluid; VDRL: venereal disease research laboratory; RPR: rapid plasm regain; Wk: week. Adapted from Ropper.

## PRION DISEASES

According to the World Health Organization, the clinical diagnostic criteria for human prion disease (HPD) include clinical findings, CSF protein markers, and electroencephalography (EEG). UK and European clinical diagnostic criteria include a combination of clinical findings, 14-3-3 protein in the CSF, magnetic resonance imaging-diffusion-weighted imaging (MRI-DWI), and EEG. CSF biomarkers determination is critical to diagnose accurately different demencial diseases. CSF biomarkers are investigated using a biochemical approach or the protein amplification methods that utilize the unique properties of prion proteins and the ability of PrP^Sc^to induce a conformational change. The biochemical markers include the 14-3-3 (ELISA or Western Blot techniques) and total tau proteins of the CSF. The sensitivity and specificity of 14-3-3γ ELISA vary from 80% to 90% and from 70% to 92% respectively, while for high levels of CSF Tau protein in HPD sensitivity varies from 84% to 96% and specificity from 30% to 98%. Other CSF biomarkers, like neuron specific enolase (NSE), S-100 protein and neurofilaments (NfL), have been studied. The sensitivities and specificities of NSE and S-100 protein are inferior to those of other biomarkers, but NfL appears as an excellent and accurate diagnostic marker in distinguishing healthy controls from patients with sporadic Creutzfeldt-Jakob disease (sCJD) presenting sensitivity between 90% and 96% and specificity between 80%-85%^22^ ^,^ ^23^.

The protein amplification method is based on the ability of PrP^Sc^to induce a conformational change continuously in prion protein thus, a small amount of PrP^Sc^found in organs, tissues, and body fluids in patients with HPD can be amplified to a point where they are detectable by conventional laboratory techniques. The protein amplification methods include the protein misfolding cyclic amplification assay (PMCA) and real-time quaking-induced conversion (RT-QuIC) assay. The RT-QuIC analysis of the CSF has been proved to be a highly sensitive and specific test for identifying sporadic HPD forms; for this reason, it is now included in the diagnostic criteria. RT-QuIC sensitivity varies between 77% and 100% and specificity rounds 100%.^22^ More recently, a second a second-generation QuIC was developed. The difference between the first- and second-generation QuIC is temperature and the use of recombinant prion protein as the seed, that is, human prion protein and hamster prion protein, respectively The assay developed by us is called first-generation QuIC, and the assay developed in Europe and the United States is called second-generation QuIC. The difference between the first- and second-generation QuIC is temperature and the use of recombinant prion protein as the seed, that is, human prion protein and hamster prion protein, respectively^23^.

In conclusion, CSF analysis is one of the most important tools in the diagnostic of central nervous system infections. It is essential to make a diagnosis of acute bacterial meningitis and leukocyte count still remains the most effective predictor of acute bacterial meningitis over newer models. In addition to the discovery of increasingly specific biochemical markers, molecular methods have been developed ensuring greater sensitivity, reducing time to diagnosis, time to appropriate anti-infective treatment (mostly empirical treatment discontinuation or de-escalation), hospital stay, and, consequently, management cost. More recently, metagenomic next-generation sequencing of CSF has the potential to identify a broad range of pathogens in a single test, allowing, despite the still high cost, greater diagnostic possibilities.
